# Salivary Indigestion

**Published:** 1895-04

**Authors:** 


### SALIVARY INDIGESTION.

A curious illustration of how scientific discovery sometimes hinders progress, is afforded by the erroneous views which have for some years prevailed in relation to the value of the saliva as a digestive agent. More than sixty years ago Leuchs discovered the fact that the saliva possesses the remarkable property of converting starch into sugar. Later investigators found that this property of the saliva is so active that if the sugar is removed by osmosis as rapidly as it is formed, a very small amount of saliva is capable of converting an almost indefinite amount of sugar. The subsequent discovery that the ptyalin, or animal diastase, as it was termed by Mialhe, required an alkaline or neutral medium to enable it to exercise its properties, led physiologists, almost without exception, to the conclusion that although possessing powerful digestive properties, the saliva is really of very little practical value as a digestive agent, on account of the acid character of the gastric juice, it being supposed that the saliva becomes inactive as soon as it reaches the stomach.

The brief period during which the food remains in the mouth is certainly wholly insufficient to enable the saliva to make any very considerable progress in the digestion of the starch which constitutes about one-half the weight of all foods of vegetable origin ; and if it were true that the saliva in entering the stomach is immediately neutralized by the gastric juice so that its action ceases, it might properly be regarded as of no value except as an aid to the mechanical disintegration of the food. This theory, however, rests upon a false foundation. Chittenden and others have shown that the digestive powers of the ptyalin of the saliva increases up to the point of actual neutrality—in other words is more active in a neutral than in an alkaline solution.

Hayem and Winter have shown that free hydrochloric acid makes its appearance in the stomach fluid only at the end of thirty minutes after the beginning of the digestive process. This gives ample time for the saliva to do its work. That the saliva actually does an enormous amount of work in the stomach under favorable conditions, that is when a sufficient amount of saliva has been mingled with the food by mastication, we have demonstrated by actual analysis in a great number of cases. During the last three years more than four thousand analyses of stomach fluid have been made in the physiological laboratory under the writer's charge, in connection with the Battle Creek Sanitarium. In each case a careful determination has been made in reference to the action of the saliva upon starch, and with the result that in 14 per cent. of the cases examined, the reaction of Lugol's solution has shown complete conversion of the starch. In 85 per cent. the starch has been partially converted. In only 17 per cent. has the blue reaction been observed, indicating little or no action upon the starch. It is more than probable that hasty mastication is one of the principal causes of dyspepsia in Americans. The gastric juice cannot act upon the starch ; it can only act upon gluten and other nitrogenous elements of bread and other cereal foods after these elements have been set free by the action of the saliva upon the starch which constitutes the greater bulk of these food substances.

This neglect of mastication and resulting salivary indigestion, explains the enormous demand for malt preparations (we do not refer to beer, which is worthless as a digestive agent) which has sprung up within the last few years. The product of malt digestion, or maltose, is precisely the same as that of salivary digestion, the action of the saliva upon the starch resulting in the production, not of glucose, as was formerly supposed, but of maltose.

During the half or three-quarters of an hour which intervenes between the swallowing of the food and the production of a degree of acidity sufficient to prevent the action upon the starch through the appearance of free hyrochloric acid, very active conversion of starch is taking place. If the food has been thoroughly masticated, so that it is broken up into fine particles, thus also ensuring an admixture of an abundant quantity of saliva, the great share of the starch elements of the food will be rendered soluble by conversion into dextrine, even if not completely converted into sugar, thus setting free the nitrogenous elements, which may be acted upon by the gastric juice in their turn.

It must not be forgotten, also, that the saliva is a most active peptogen; that is, the presence of the saliva in the stomach, in connection with the food, stimulates glandular activity on the part of the stomach whereby an active and abundant supply of gastric juice is produced.

Another cause of salivary indigestion which we should mention, is the abundant use of sweets. In order that the saliva shall exercise its properties efficiently, it is necessary that it should act in a suitable medium. A temperature of 100° and an alkaline or neutral reaction are necessary for prompt and vigorous action on the part of the saliva upon the farinaceous elements of food. A low temperature hinders this action, and acidity stops it altogether. The presence of a large amount of sugar also hinders the action of the saliva.

It is thus evident that the copious drinking of cold water, or the taking of ice foods in connection with meals, is a means of producing salivary indigestion. The free use of strong acids, such as vinegar, in connection with cereal foods, is equally objectionable. Nothing could be more absurd than the combination of strong acids with vegetable elements, as in pickles. This is probably the reason why many persons find themselves unable to use acid fruits without fermentation. The acidity may be sufficient to neutralize the action of the saliva upon the starch.

Evidently it is not only physiologically absurd to add sugar to farinaceous foods, since the starch, which composes one-half the weight of these foods, is all converted into sugar in the process of digestion, but the practice is also highly injurious, since it prevents the normal action of the saliva upon the starch. In this way, sugar preserves, sweet sauces, confectionery, ice-cream, cakes, and other sweets, are in the highest degree conducive to salivary indigestion.

In the use of candy and glucose syrups, the injurious effects are intensified by the fact that chemically prepared glucose is not a physiological sugar. The fact that glucose may be produced by the action of sulphuric acid upon starch was discovered by Kirchhoff early in the present century. Dubrunfaut first, and later, Morris, O'Sullivan, and others have shown that the maltose produced by the saliva and the pancreatic juice, and the glucose produced by the action of sulphuric acid upon starch, are two entirely different products. Glucose is sweet, and, from a chemical standpoint, is a sugar, but is not the sugar produced by the action of either the saliva or the pancreatic juice upon the starch : hence it cannot be considered a substitute for these sugars. Glucose ferments with very great facility, whereas maltose does not so readily undegro fermentation. The same is true also of the sucrose, or cane sugar, which is formed in the natural process of plant growth. The wisdom of this arrangement is readily appreciated in view of the extremely favorable conditions for fermentations which exist in the stomach. Yeast and microbes of various sorts are always present in the stomach, where is found the degree of warmth and moisture necessary to facilitate the action of vegetable ferments upon saccharine substances. Nature has so arranged it that the saccharine substances taken into the stomach or produced in the stomach and in the digestive process shall not be readily fermentable. Both cane sugar and maltose are changed into glucose before absorption. But this change takes place apparently during the passage of the saccharine substances through the mucous membrane of the small intestines, since this part of the alimentary canal alone is possessed of the power of producing glucose.

The consequences of salivary indigestion are : Acid fermentations, heartburn, stomach and intestinal colic, dilatation of the stomach, catarrh of the stomach, and many evil effects arising from these conditions.

The remedy for salivary indigestion consists in prohibiting sweets, ices, and soft foods, and requiring patients to masticate thoroughly every particle of food swallowed. In many cases it is well to aid the process of salivary digestion by exposing the cereal food substances to the prolonged action of heat, thereby converting the starch into dextrine, rendering it more readily soluble, and hence more readily acted upon by the saliva. Granola and zwieback are invaluable articles of food for use in cases of this sort. The malt preparations are useful as palliatives in some cases, but it should be remembered that it is wrong to become dependent upon any artificial digestive agent. —*Modern Medicine and Bacteriological Review, Editorial.*

